# Colour polymorphic lures exploit innate preferences for spectral versus luminance cues in dipteran prey

**DOI:** 10.1186/s12862-017-1043-7

**Published:** 2017-08-14

**Authors:** Thomas E. White, Darrell J. Kemp

**Affiliations:** Department of Biological Science, Macquarie University, Sydney, 2109 Australia

**Keywords:** Deception, Communication, Visual ecology, Fly, Spider, Signalling, Polymorphism

## Abstract

**Background:**

Theory predicts that colour polymorphism may be favored by variation in the visual context under which signals are perceived. The context encompasses all environmental determinants of light availability and propagation, but also the dynamics of perception in receivers. Color vision involves the neural separation of information into spectral versus luminance channels, which often differentially guide specific tasks. Here we explicitly tested whether this discrete perceptual basis contributes to the maintenance of polymorphism in a prey-luring system. The orb-weaving spider *Gasteracantha fornicata* is known to attract a broad community of primarily dipteran prey due to their conspicuous banded dorsal signal. They occur in two morphs (“white” and “yellow”) which should, respectively, generate greater luminance and color contrast in the dipteran eye. Given that arthropods often rely upon luminance-versus-spectral cues for relatively small-versus-large stimulus detection, we predicted a switch in relative attractiveness among morphs according to apparent spider size.

**Results:**

Our experimental tests used colour-naïve individuals of two known prey species (*Drosophila hydei* and *Musca domestica*) in replicate Y-maze choice trials designed to manipulate the apparent size of spider models via the distance at which they are viewed. Initial trials confirmed that flies were attracted to each *G. fornicata* morph in single presentations. When given a simultaneous choice between morphs against a viewing background typical of those encountered in nature, flies exhibited no preference regardless of the visual angle subtended by models. However, when backgrounds were adjusted to nearer the extremes of those of each morph in the wild, flies were more attracted by white morphs when presented at longer range (consistent with a reliance on achromatic cues), yet were unbiased in their close-range choice.

**Conclusion:**

While not fully consistent with predictions (given the absence of a differential preference for stimuli at close range), our results demonstrate an effect of apparent stimulus size upon relative morph attractiveness in the direction anticipated from present knowledge of fly visual ecology. This implies the potential tuning of *G. fornicata* morph signal structure according to a perceptual feature that is likely common across their breadth of arthropod prey, and complements recent observational work in suggesting a candidate mechanism for the maintenance of deceptive polymorphism through the exploitation of different visual channels in prey.

**Electronic supplementary material:**

The online version of this article (doi:10.1186/s12862-017-1043-7) contains supplementary material, which is available to authorized users.

## Background

The maintenance of intraspecific diversity is of enduring interest in evolutionary biology. Colour polymorphisms have proven valuable for examining the causes and consequences of conspicuous variation [1–4]. Research on classic cryptic [5, 6], sexual [7], and mimicry [8–10] systems has done much to identify the selective processes that mediate the coexistence of discrete morphs. Divergent selection is one factor that has been implicated in the maintenance of stable polymorphisms (e.g. the *Papilio dardanus* and coral snake mimicry systems; [8, 11]).

In visual communication systems, the effectiveness of signals is shaped by the interaction between viewing environments and the perceptual systems of receivers [12, 13]. Variation in environmental features such as the spectral quality and/or intensity of available light will modify the appearance of a given signal design [12]. When unpredictable, variation of this nature contributes ‘noise’ to signaling systems, which is a basic challenge for effective communication. In response, selection may favour more precise behavioural delivery of signals [14, 15], restricting displays only to favourable environmental conditions [16], or actively modifying viewing environments [17, 18].

A general possibility, formalised in sensory drive theory, is that variation in the components of signalling systems may favour signal polymorphism by generating distinct signalling ‘niches’ [19, 20]. This may be particularly applicable  in static signalling contexts, such as prey attraction by sit-and-wait predators. In these cases, encounters with receivers are largely unpredictable—that is, they occur under variable visual conditions—and behavioural fine-tuning of signal delivery is largely unavailable. The signaling niche hypothesis is supported by evidence from sexual and aposematic systems in which selection for effective communication in heterogeneous environments has driven the diversification of visual signals [5, 21–25]. For example, recent experimental work has shown that the polymorphic warning colours of dyeing poison frogs (*Dendrobates tinctorius*) are differentially detectable to vertebrate predators under varied lighting conditions [25]. This variation in ambient light occurs across fine spatial and temporal scales throughout the frogs’ natural forest habitat, which may favour the maintenance of signal polymorphism. Such work has, however, almost exclusively focused on abiotic sources of variation—such as light environments and viewing backgrounds—rather than potential biotic sources, including receiver perception [26].

Visual systems are typically equipped to extract both colour and luminance information from scenes, which often involves the use of specialised neural architectures [27]. Birds, for example, process colour information by comparing the relative stimulation among sets of opposing photoreceptors (i.e., a cone-based opponency system), but largely rely on broadly tuned double-cones to perceive luminance (reviewed in [28]). Honeybees (*Apis mellifera*), in contrast, have three photoreceptor sub-types that are partly responsible for both tasks. Colour vision is based on the comparison of outputs from all three receptors, while luminance-based tasks are likely guided by outputs from the long-wavelength ‘green’ receptor [29–31]. Animals often (though not exclusively) use colour and luminance information to guide distinct visual tasks [27, 32–34]. Colour information is typically relied upon for the task of large-object detection and classification, for example, while luminance information may inform motion vision, as well as the perception of form and texture at a distance [27, 31–33, 35, 36; see further specific discussion in the methods].

From the perspective of signallers, the partitioning of visual information may offer alternate routes to visual conspicuousness through the enhancement of colour- or luminance-based contrast. This may be particularly true in noisy environments, wherein viewing conditions—and hence the demands on a visual system—can shift rapidly over small temporal and spatial scales [12]. The conspicuous and often polymorphic visual lures of orb-web spiders (reviewed in [26]) offer excellent opportunities for examining this hypothesis. Their deceptive signals are statically displayed in visually noisy forest environments [12, 35], and we know little about the adaptive maintenance of polymorphism in this context.

**Fig. 1:**
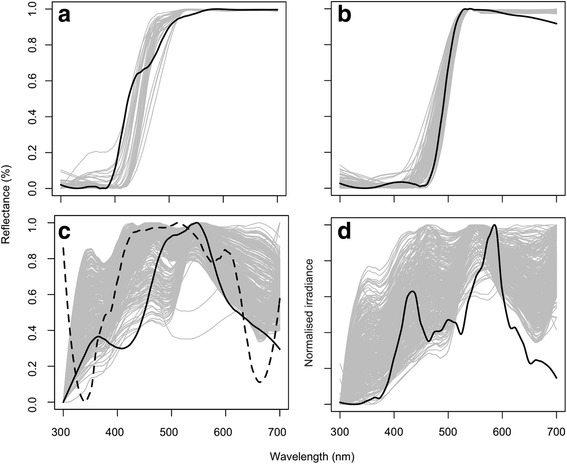
The spectra of stimuli used in choice assays (black lines) and their natural equivalents (grey lines, from [35]); **a** white *G. fornicata*, **b** yellow *G. fornicata*, **c** backgrounds, both “average” (experiments 1–3, solid line) and “augmented” (experiment 4, dashed line) and **d** illuminant. Although normalized here for ease of comparison, all stimuli were matched to natural sources considering both spectral intensity (‘*brightness*’) and shape (‘*colour*’). Note that since flies are largely insensitive to wavelengths above 600 nm, all perceptually relevant spectral variation is limited to the 300–600 nm range

*Gasteracantha fornicata* is an orb-web spider, distributed throughout tropical and sub-tropical forests in Australasia, that uses conspicuous dorsal colouration to lure prey [35–37]. Females are stably polymorphic, exhibiting UV-negative ‘white’ or ‘yellow’ bands (as perceived by humans) against a black outline. The species exhibits a stable cline in morph frequencies along their distribution, with the white morph prevailing at their northernmost range limit, and the yellow morph at their southernmost [examined in 38]. As is common among orb-web spiders, *G. fornicata* are strongly sexually dimorphic, with small, cryptic, mobile males (that are therefore of limited interest in a visual signalling context). Recent field-based research on females of the species suggested that yellow morphs most effectively attracted prey under conditions in which they presented greater chromatic contrast against viewing backgrounds, while white morphs were more attractive when presenting a relatively greater achromatic contrast (across all potential viewing scenarios; [35]). These results are consistent with the hypothesis that their deceptive signals are differentially tuned to enhance chromatic (among yellow morphs) or achromatic (among white morphs) background contrast, respectively, from the perspective of their primarily dipteran prey (with a likely-minimal influence of avian predation upon signal evolution; [35]). Here we sought to experimentally test this hypothesis. We predicted that dipteran viewers should prefer white-banded morphs at longer viewing distances when the apparent size of spiders is relatively small (and viewers’ visual systems are thus assumed to be prioritizing luminance cues, discussed further below). This follows from the fact that white morphs will, by virtue of their broader spectral reflectance band (Fig. 1a, b), generate greater positive luminance contrast against typical visual backgrounds. Conversely, we predicted that dipteran viewers should favour yellow morphs at shorter viewing distances when the apparent size of spiders is relatively greater (and viewers are thus assumed to be guided by chromatic cues), given the documented preference for yellow stimuli among flies [38–42]. We conceived an approach for testing these predictions that exploited the differential use of colour-versus-luminance information by flies’ visual systems according to the apparent size of the stimulus.

## Methods

### Overview

We conducted a series of experiments using colour-naive *Drosophila hydei* and *Musca domestica* in a common “y-maze” apparatus (Additional file 1: Figure S1). This apparatus provided test subjects with the opportunity to orient towards one of two destinations according to the visual stimulus being presented in either arm (as broadly equivalent to a “binary choice test”). In order to test whether *G. fornicata* morphs are differentially attractive depending on the visual channel being prioritised, we sought to manipulate the visual channel being relied upon by varying the apparent size of model spiders presented to dipteran viewers (i.e. the visual angle subtended by models at the point at which they were first viewed; Additional file 1: Figure S1). This manipulation was informed by research across taxa (as outlined above) suggesting that a general feature of diurnal visual systems, including birds, bees, and primates, is the differential use of colour and luminance information depending on the apparent size of the stimulus. Achromatic cues are often used for the detection of stimuli subtending small visual angles, while stimuli spanning relatively larger visual angles are primarily identified using chromatic cues [31–33, 43, 44]. Given the weight of this assumption, we first conducted a pilot experiment to test whether innate preferences for colour and luminance cues in flies may alternate as a function of apparent stimulus size. We used a modified version of the choice assay outlined below, and our results are broadly consistent with the notion that *D. hydei* and *M. domestica* innately prefer achromatic cues at relatively small visual angles (10°), and chromatic cues at larger angles (50°; see Additional file 1 information for full details).

### Animal rearing

All *D. hydei* used in the experiments were first- to third- generation offspring of wild caught adults, and we did not differentiate sexes. We stored wild-caught adults in in 750 ml Pyrex glass storage bottles, with 100 ml of colourless food-culture lining the base. The culture was a 4.0:1.0:1.0:0.1 ratio of instant mashed potato, icing sugar, water, and yeast, and we removed F0 adults within 24 h of laying eggs. We purchased *M. domestica* as pupae from a commercial breeder (Pisces Enterprises, Queensland, Australia), and stored them in 750 ml Pyrex glass storage bottles until eclosion, with unlimited access to sugar-solution. We reared all flies in a temperature controlled laboratory (25 ± 2 °C) with a 14:10 L: D photoperiod, and used all individuals in experimental trials within four days of eclosion.

### Experimental stimuli and environment

We conducted all experiments using the y-maze apparatus situated in a temperature-controlled laboratory (25 ± 1 °C) at Macquarie University, Australia. The maze consisted of a flight cage (400 × 300 × 300 mm; length, width, height) attached to two arms (1100 × 250 × 300 mm) at 90° (Additional file 1: Figure S1). The top of the maze was covered with a single sheet of clear screen (Rosco “Supergel 00”), which evenly transmits >95% of wavelengths in the 300–700 nm range. We illuminated the maze with a 400 W Sylvania metalarc-halogen light diffused with a single sheet of ultraviolet-transmitting Rosco 216 diffusion screen. This imitates natural overcast conditions (sensu [45, 46]) and represents a reasonable approximation of of possible illumination conditions under which *G. fornicata* are viewed in the wild (Fig. 1).

**Fig. 2:**
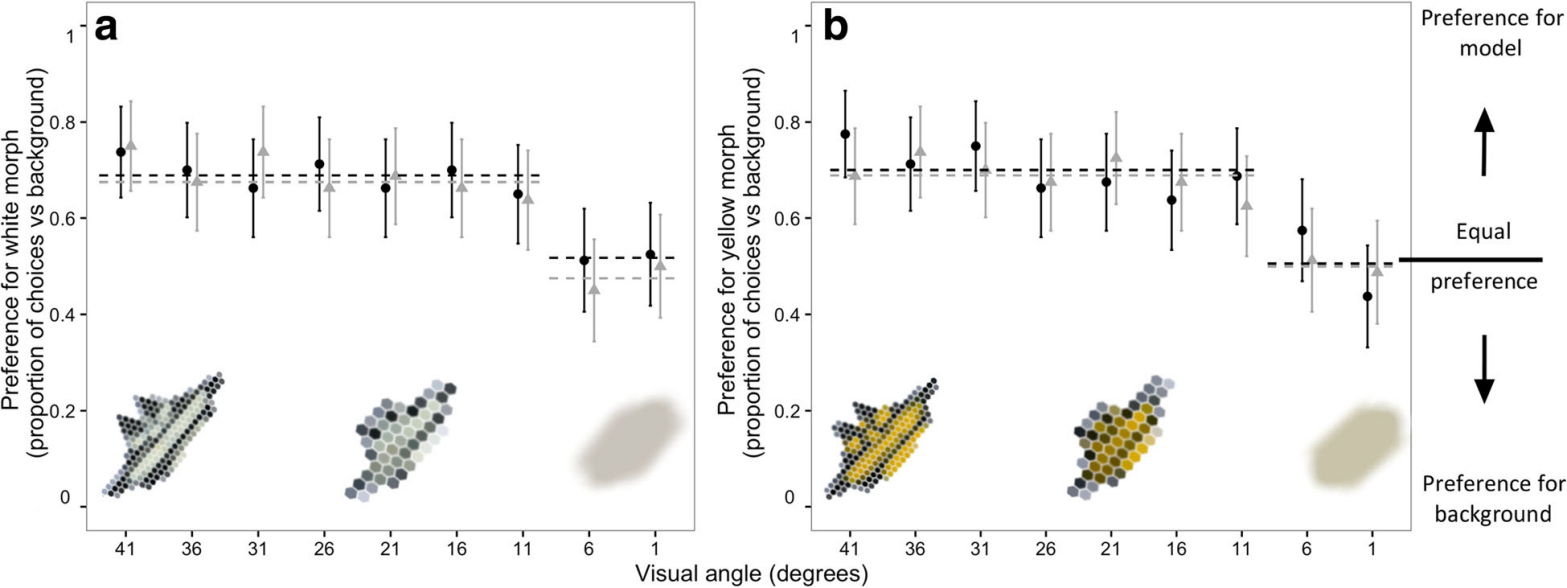
The results of experiments one and two, showing the proportion of choices (mean ± 95% C.I., *n* = 80 per visual angle category) by *D. hydei* (dark points and line) and *M. domestica* (light points and line) for **a** white spider model and **b** yellow spider model, each versus a plain “average” background, as a function of the visual angle subtended by models. Dashed lines indicate mean choice frequencies within homogenous sets, as determined by a-posteriori simultaneous G-tests (Table 2). Stylized spider model images (inset) are shown to loosely approximate how the banded pattern would appear across this range given the known limits of fly spatial (but not spectral) resolution

We built models of *G. fornicata* (12 × 30 mm, length x width; Fig. 2 inset) using 1 mm black cardboard (Elle Card, ‘Black’). The stripes of white morphs were painted with a 1.0:0.2 mix of Jo Sonjas ‘warm white’ and Reeves ‘pale lemon yellow’ acrylic paints, and yellow models with Derivan Matisse Yellow-Mid AZO Series 2. All models were covered with a single piece of clear, ultraviolet-absorbing Rosco cinegel 3114, to remove all ultraviolet reflectance and to imitate the glossiness of *G. fornicata*. In experiments one to three, we presented models against card that was spectrally matched to resemble an ‘average’ background as measured for *G. fornicata* in the wild (Fig. 1c; Elle Card, ‘Verdone’). In experiment 4, discussed further below, we displayed models against augmented backgrounds (Elle Card, ‘Storm’) that enhanced the colour and luminance contrasts of yellow and white morphs, respectively.

We ensured that models, backgrounds, and illuminants fell within natural ranges of intensity and colour (Fig. 1) by matching them to spectral measurements obtained previously for spiders at sites in North Queensland, Australia [35]. All spectral measurements were taken using a JAZ EL-200 portable spectrometer fitted with a cosine-corrected irradiance module, and a PX-2 pulsed xenon light source (OceanOptics Ltd., Dunedin, USA). Reflectance was quantified using a “beam measurement” set-up (boxcar width = 10, integration time = 100 ms, scans to average = 10), as extensively used to measure *G. fornicata* specimens [35, 37]. This consisted of separate light source (PX-2) and collector probes situated at 90° and 45° relative to the sample plane, with a 99% diffuse “spectralon” reflectance standard used for calibration (Labsphere, New Hampshire) between samples. We captured the spectra via OceanOptics SpectraSuite software (ver. 1.6.0_11), and processed them using the development version of the package ‘pavo’ (ver. 1.0; [47]) for R (ver. 3.2.5; [48]). Following auto-calibration for ambient intensity, we recorded vector irradiance at a point immediately above and parallel to the top of the y-maze.

### Choice assays

We conducted four separate experiments, varying the stimuli and backgrounds presented to receivers in each. In all experiments, spider models were presented in the centre of a 250 × 300 mm cardboard background (see further below for details). We varied the distance between the stimuli and the maze’s ‘choice point’ (D in Additional file 1: Figure S1) to control the visual angle subtended by the models (calculated using the left-right axis of model spiders) in nine increments: 1°, 6°, 11°, 16°, 21°, 26°, 31°, 36°, and 41°.

We initiated trials by introducing each colour-naive subject into an enclosed compartment at the head of the Y-maze. After three minutes of visual adaptation time we opened the entrance to the decision chamber and the flies, upon entering, were able to simultaneously view both arms. A decision was registered when the subject crossed the ‘choice’ line at the entrance to a maze arm (Additional file 1: Figure S1). We discarded individuals that did not leave the flight chamber within 10 minutes of the entrance opening (ca. 14% of *M. domestica*, and 6% of *D. hydei*). Model stimuli were randomly assigned across arms of the Y-maze, and the ordering of visual angles at which models were presented was randomised at the beginning of each experiment. We recorded the responses of 80 individuals of both *D. hydei* and *M. domestica* at each visual angle, and did not re-use individuals; each fly made a single choice. We therefore used 80 individuals at each of nine apparent model sizes, totalling 720 individuals/choices per species in each experiment.

### Experiments 1 and 2: Spider models versus plain backgrounds

In experiments one and two we offered flies a choice between either a yellow (experiment 1) or white (experiment 2) spider model versus a background with no spider model. The card background in these experiments approximated the visual average for this species as measured in extensive field sampling [35], that is, the averaged spectrum presented by visual backgrounds of *G. fornicata* in the wild. The purpose of this was twofold. First, it allowed us to assess the baseline attractiveness (if any) of each *G. fornicata* morph to naive receivers, as a function of apparent model size. Second, it allowed us to determine the point at which receivers no longer responded to the models, which we expected to occur at or near visual angles on the order of the spacing of individual ommatidia (ca. 1–11°; [49–52]).

### Experiments 3 and 4: Yellow versus white spider models against “average” versus “augmented” backgrounds

These two experiments aimed to test the hypothesis that flies should orient preferentially towards yellow morphs when relying on chromatic cues, and white morphs when guided by achromatic cues. If *M. domestica* and *D. hydei* alternate their reliance on colour-versus-luminance information as a function of apparent stimulus size (as discussed above and supported by the pilot experiment; see Additional file 1), this hypothesis predicts that subjects will be attracted to yellow morphs at relatively larger visual angles but shift towards white morphs at relatively smaller visual angles (across both experiments).

In both experiments we presented individual flies with the simultaneous choice of a yellow versus white spider model presented in each arm of the Y-maze. In experiment three, spider models were situated against the same average background used in experiments 1–2. In experiment four, we varied this to a relatively dull, less saturated background. While still within the natural range of signalling conditions (Fig. 1c), this background differentially enhanced spider/background colour and luminance contrasts for yellow and white morphs, respectively. This meant that yellow models were likely more ‘colourful’ and white models more ‘luminant’ relative to their predicted difference in appearance in experiment three (Table 1 and Additional file 1). That is, experiment four introduced greater *differential* (though not absolute; Table 1) variation in colour and luminance contrasts between morphs, while remaining within the natural, and perceptible, range of signal/background contrasts encountered by dipteran viewers in the wild [35, 42]. The rationale for this manipulation follows from observational data for when *G. fornicata* actually capture prey in nature [35]. These data indicated that yellow morphs caught most prey under visual scenarios when they presented a more ‘colourful’ signal, while white morphs were more attractive when presenting a relatively more ‘luminant’ signal (as mediated by temporal and spatial variation in viewing backgrounds and light conditions, as well as receiver physiology; [35]). Our use of an augmented background therefore aspired to introduce a more pronounced, yet functionally relevant, degree of variation in signal/background contrast, beyond the levels achieved using the average of all possible natural backgrounds as in experiment 3.

**Table 1:** The broadly estimated chromatic (unitless) and achromatic (Michelson) contrast of model spider morphs against their backgrounds in all experiments, as modelled according to the visual systems of *D. melanogaster* and *M. domestica* (Additional file 1)

		Experiments 1–3		Experiment 4	
viewer	model	chromatic	achromatic	chromatic	achromatic
*D. melanogaster*	white	0.35	0.67	0.37	0.39
	yellow	0.38	0.42	0.43	0.21
*M. domestica*	white	0.37	0.71	0.40	0.45
	yellow	0.39	0.45	0.48	0.23

### Data analysis

We used goodness of fit tests (G-tests) of homogeneity throughout to test for deviations from equal-choice frequency as a function of the visual angle at which models were presented. When significant heterogeneity was detected, we used a-posteriori simultaneous G-tests for homogeneity to identify which sets of responses differed from one another [53]. Briefly, this entailed a stepwise testing procedure wherein the response data, beginning with the largest visual angle category (41°), were cumulatively grouped into sets and tested for homogeneity (against a critical G-value adjusted for multiple comparisons). In experiment 1, for example, the 41° and 36° response-categories were first tested, then the 31° data were added to this set and retested, and so on. This continued until a maximally non-significant set was found, at which point we repeated the procedure in reverse, moving from the smallest-to-largest visual angle category. This procedure allowed us to identify any significant shifts in the attractiveness of model spiders as a function of visual cues being prioritised by receivers’ visual systems (e.g. Fig. 2). In experiments one and two, we expected at least one change-point corresponding to the apparent model size at which flies no longer respond to stimuli. In experiments three and four, our hypothesis (as discussed above) predicts that yellow morphs should be relatively more attractive at larger apparent sizes, while white morphs should be relatively more attractive at smaller sizes, though the precise point at which this should occur cannot be predicted a-priori. We excluded tests at the 1° and 6° visual angles in experiments three and four because the first two experiments established no effective response to stimuli subtending less than 11°.

## Results

### Experiments 1 and 2: Spider models versus plain backgrounds

Relative to a no-stimulus alternative, white *G. fornicata* morphs proved attractive to both *D. hydei* (G_8_ = 17.76, *P* = 0.023) and *M. domestica* (G_8_ = 27.97, *P* < 0.001) across a range of apparent model sizes (Fig. 2a). A-posteriori simultaneous tests identified two distinct groupings of responses, corresponding to visual angles of 1–6°, versus 11–41°. Subjects were consistently attracted to models that subtended visual angles between 11 and 41° (at ca. 70% response rate), but this fell to equality when presented at apparent sizes of 6° or less (Fig. 2a; Table 2).

**Table 2:** Summary of a-posteriori simultaneous tests for homogeneity, from experiments 1 and 2 in which flies were offered the choice of spider models were presented versus a plain background

		Experiment 1 (White model vs plain bkg)				Experiment 2 (Yellow model vs plain bkg)			
Simultaneous test set (visual angle category)		*Drosophila hydei*		*Musca domestica*		*Drosophila hydei*		*Musca domestica*	
Set 1	Set 2	G	P	G	P	G	P	G	P
41-36^O^		0.27	0.60	1.10	0.29	0.82	0.36	0.49	0.48
41-31^O^		1.07	0.58	1.27	0.53	0.83	0.66	0.53	0.77
41-26^O^		1.12	0.77	2.23	0.53	2.86	0.41	0.84	0.84
41-21^O^		1.61	0.81	2.34	0.67	3.63	0.46	1.04	0.90
41-16^O^		1.62	0.90	2.83	0.73	5.50	0.36	1.32	0.93
41-11^O^		2.28	0.89	3.90	0.69	5.56	0.47	3.07	0.80
41-6^O^		14.58	0.05*	20.55	< 0.01*	28.69	< 0.01*	14.46	0.04*
	1-6^O^	0.03	0.87	0.40	0.53	3.04	0.08	0.10	0.75
	1-11^O^	8.63	0.03*	6.12	0.04*	10.31	< 0.01*	7.93	0.04*

‘Visual angle’ describes the apparent size of model spiders as viewed by flies at the choice point in a Y-maze apparatus (Additional file 1: Figure S1)

Asterisks indicate significant heterogeneity within a set, with maximally non-significant sets immediately above

We found qualitatively identical results in experiment two. Yellow *G. fornicata* were attractive to *D. hydei* (G_8_ = 28.63, *P* < 0.001) and *M. domestica* (G_8_ = 22.11, *P* = 0.005), but only once they subtended visual angles >11° across a range of apparent model sizes (Fig. 2b). This too was consistent at presentation angles of 11–41°, and fell to equality at 6° or less (Fig. 2b; Table 2).

### Experiments 3 and 4: Yellow versus white spider models against “average” versus “augmented” backgrounds

**Fig. 3:**
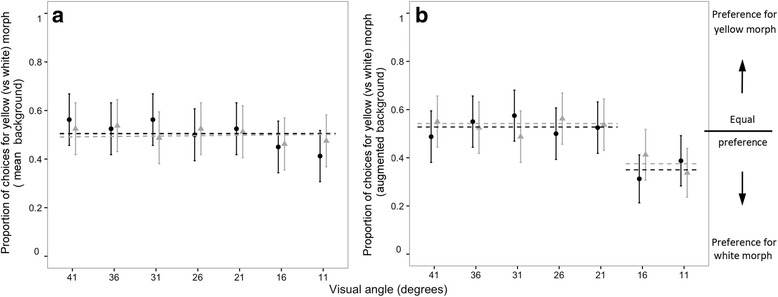
The results of experiments three and four, showing the proportion of choices (mean ± 95% C.I., *n* = 80 per visual angle category) by *D. hydei* (*dark points and line*) and *M. domestica* (light points and line) for yellow-versus-white spider models presented against (**a**) “average” backgrounds and (**b**) “augmented” backgrounds. Dashed lines in indicate mean choice frequencies within homogenous sets, as determined by a-posteriori simultaneous G-tests (Table 3)

When simultaneously presented against average backgrounds, each *G. fornicata* morph proved equally attractive to *D. hydei* (G_6_ = 6.10, *P* = 0.411) and *M. domestica* (G_6_ = 1.57, *P* = 0.955) across the 11–41° range (with a possible linear trend; Fig. 3a). When simultaneously presented with against an augmented background, however, the relative attractiveness of morphs was heterogeneous for both *D. hydei* (G_6_ = 17.27, *P* = 0.008) and *M. domestica* (G_6_ = 13.47, *P* = 0.036; Fig. 3b). A-posteriori simultaneous tests revealed a single shift in receiver responses; morphs were equally attractive when subtending visual angles of 41–21°, while white spiders were distinctly more attractive at apparent sizes of 11–16° (Fig. 3b, Table 3).

**Table 3:** Summary of a-posteriori simultaneous tests for homogeneity, from experiment 4 in which flies were offered the simultaneous choice of yellow or white-banded spider models, presented versus an “augmented” background

Simultaneous test set (visual angle category)		*Drosophila hydei*		*Musca domestica*	
Set 1	Set 2	G	P	G	P
41-36^O^		0.62	0.43	0.10	0.75
41-31^O^		1.31	0.52	0.63	0.73
41-26^O^		1.65	0.65	1.05	0.79
41-21^O^		1.65	0.80	1.06	0.9
41-16^O^		14.26	0.02*	13.47	0.04*
	11-16^O^	0.99	0.32	0.96	0.33
	11-21^O^	7.70	0.02*	6.69	0.04*

Visual angle describes the apparent size of model spiders as viewed by flies at the choice point in a Y-maze apparatus (Additional file 1: Figure S1)

Asterisks indicate significant heterogeneity within a set, with maximally non-significant sets immediately above

## Discussion

Theory predicts that variation in selection may maintain intraspecific diversity, including polymorphism [54, 55]. Signalling environments and the perceptual systems of receivers are sources of ample variation known to drive intraspecific diversity, albeit chiefly in sexual ornaments [21, 24, 56]. Here we tested the potential for conditional use of visual information in receivers to favour colour polymorphism in a prey-lure context. We found evidence that each *G. fornicata* morph appeals to innate spectral and luminance biases in dipteran prey (which may include simple stimulus-presence; Fig. 2), at least relative to a non-stimulus background. However, the central prediction—that yellow and white morphs should differentially exploit colour and luminance preferences, respectively—was only partly supported by our data. Although relative morph attractiveness did vary depending on the visual cues putatively prioritised by flies’ visual systems (i.e., as a function of apparent model size), this effect was conditional upon the relative magnitude of signal/background visual contrast between morphs (Fig. 3). We expand upon these results in discussion of how interactions between signal structure, signalling environments, and innate receiver biases may favour colour-lure polymorphism.

Our finding that the colour-lures of *G. fornicata* are attractive to dipteran prey (Fig. 2) is not surprising. Flies are known to orient towards yellow and non-ultraviolet-white stimuli (reviewed in [42]), and such stimuli have known efficacy across a range of prey luring taxa [36, 57–61]. Precisely which biases are exploited is, however, poorly understood. Our use of colour-naive viewers suggests that innate visual biases, at least in part, underlie the attractiveness of deceptive lures across a range apparent lure sizes. Of course we cannot be certain the innate attractiveness of *G. fornicata* models was driven by their (UV-negative) yellow or white signals specifically (since these were the only two colours tested), though these results are consistent with the known colour preferences of flies, as discussed above. Experiments one and two also indicated that the absolute strength of preference was strikingly consistent (at ca. 70%) for each morph until the apparent size of models was reduced below 11° (Fig. 2). The loss of attractiveness at smaller visual angles most likely occurred because model size approached the limits of visual resolution in both fly species [49–51]. Our results also imply that both the chromatic and achromatic components of lures are innately attractive to prey; a subtlety that cannot be informed by semi-natural experiments in comparable systems given their inherent lack of precision control over viewing conditions [36, 57, 58]. Because we only have a broad sense of when colour and luminance information might be differentially favoured by flies (see pilot experiment; Additional file 1), we cannot identify the precise conditions under which prey preferences are driven by either cue. These findings nonetheless indicate that both chromatic and achromatic components of deceptive lures are likely targeted by selection for signal efficacy.

Experiments three and four revealed a shifting and at times differential degree of attractiveness among *G. fornicata* morphs according to signalling conditions (Fig. 3). This is broadly consistent with the principles of sensory drive [20], but deviates partly from our specific predictions regarding the contextual attractiveness of yellow and white morphs. When presented against a background designed to imitate an average visual scene, morphs were equally attractive to both *D. hydei* and *M. domestica* irrespective of visual angle (Fig. 3a). When instead presented against a background that further enhanced the relative (not absolute) luminance contrast of white models and colour contrast of yellow models, white morphs proved distinctly more attractive at smaller visual angles (Fig. 3b). Such angles represent smaller stimulus sizes (indeed, nearing the apparent limit of visual resolution), and therefore typify the task of longer-range orientation when flies are thought to be guided by luminance cues [42].

We found no support, however, for the predicted greater relative attractiveness of yellow morphs at any larger visual angle (Fig. 3b). This finding has several potential (non-exclusive) explanations. One possibility is simply that neither *M. domestica* nor *D. hydei* are able to discriminate between morphs based on chromatic cues, irrespective of the backgrounds against which they are presented. While there is evidence that some flies, such as the blowfly *Lucilia* sp., have a relatively poor colour-sense [62, 63], this finding is inconsistent across fly taxa. Behavioural tests with *D. melanogaster* [64, 65], for example, suggest an ability to discriminate between stimuli based on their spectral properties, with optimal performance at the 420 and 490 nm wavelength regions (which coincide with the approximate regions of difference between *G. fornicata* morphs; Fig. 1). While the implications of these studies for our understanding of dipteran colour discrimination is limited by their use of self-luminous stimuli (discussed in [42]), it is plausible that *D. hydei* and *M. domestica* can discriminate between simultaneously presented models on the basis of colour. A related possibility is that the differential increase in chromatic contrast for yellow morphs in experiment four was too subtle to elicit the predicted increase in relative attractiveness at larger visual angles (Fig. 3b). Assessing this possibility would again require an understanding of the limits of colour discrimination in flies, as well as assays across a great chromatic span of model/background combinations. Finally, as discussed below, the equal attractiveness of morphs at larger presentation angles may reflect genuinely equivalent stimulation under the conditions tested.

Our finding that the relative attractiveness of colour-lure morphs is shaped by an interaction between signal structure, signalling conditions, and receiver visual ecology (Fig. 3) is consistent with the hypothesis that signalling ‘niches’ may emerge as product of variation in the components of communication systems [19, 20]. While the data hint at a consistently greater fitness benefit for white morphs (as they proved equally or more attractive than yellow morphs under all tested conditions; Fig. 3), our assay environments were greatly oversimplified relative to the complexity of natural conditions. The spectral quality and intensity of light, particularly in forests, regularly shifts by orders of magnitude over small spatial (e.g. under patchy canopies) and temporal (e.g. with weather conditions) scales [13, 20]. Receivers’ visual and cognitive systems are similarly changeable. Colour perception may vary with the state of adaptation in photoreceptors, and the attentiveness of receivers will shift based on internal (e.g. hunger) and external (e.g. the presence of potential mates or predators) motivations [19, 66].

The most significant unexplored axis of variation was receiver experience. Controlled behavioural experiments have shown that the use of colour and luminance cues may be relatively plastic, with the attractiveness of colourful versus luminant stimuli shifting based on the past experience of receivers [67, 68]. With specific reference to flies, Troje [62] examined the responses of naive and trained blowflies to monochromatic stimuli that differed in intensity, and found that the intensity of the stimulus drove innate, but not trained, colour preferences. An intriguing possibility in case of *G. fornicata*, then, is that while white morphs are disproportionately attractive to naive receivers guided by luminance cues (Fig. 3b), the attractiveness of yellow morphs may be more strongly determined by receivers’ learned experience with coloured stimuli. As pollinators, *M. domestica* and *D. hydei* would be exposed to rewarding flowers over the course of their lifetime. Indeed, the signals of many polymorphic spiders, including *G. fornicata*, are known to resemble sympatric flowers [69], which may predictably shape the attractiveness of *G. forniatica* morphs depending on the structure of local floral communities. Determining the relative contributions of innate (colour-naive), spontaneous (colour-experienced, but untrained) and learned (plastic) biases in the maintenance of lure-polymorphism would require experiments across a range of receiver experience, for which the highly tractable orb-web spiders are exceptionally well suited.

## Conclusions

Visual signalling systems contain ample sources of variation that may contribute to the maintenance of colour polymorphism. While the role of abiotic variation in establishing ‘signalling niches’ is well supported [21–25], the question of whether perceptual variation may contribute in a similar manner is largely unexamined. In the first direct test of the possibility, our results implicate the conditional prioritisation of visual information in receivers as a likely contributing factor to the maintenance of colour polymorphism in a deceptive context. While not fully consistent with predictions, our results demonstrate an effect of apparent stimulus size upon relative morph attractiveness in the direction anticipated from present knowledge of fly visual ecology [42]. The discrete signals of *G. fornicata* may thus be tuned according to a perceptual feature that is common across their breadth of arthropod prey. This complements recent observational work [35] in suggesting an unappreciated avenue for the maintenance of polymorphism through the exploitation of different visual channels in receivers.

## Additional file

Additional file 1: Additional experiment: details the methods of the additional experiment. **Figure S1** depicts the Y maze apperatus for choice assays. **Figure S2 **shows the reflectance spectra of stimuli used in the additional choice experiment. **Figure S3** presents the results of the supplementary experiment. **Table S1** denotes the approximate chromatic (unitless) and achromatic (Michelson) target/background contrast of model ‘colourful and ‘luminant’ stimuli from Additional experiment S1, as modelled according to the visual systems of *D.melanogaster* and *M. domestica*. (PDF 329 kb)

## References

[1] White TE, Kemp DJ (2016). Colour polymorphism. Curr Biol.

[2] Gray SM, McKinnon JS (2007). Linking color polymorphism maintenance and speciation. TREE.

[3] McLean CA, Stuart-Fox D (2014). Geographic variation in animal colour polymorphisms and its role in speciation. Biol Rev.

[4] Ford EB (1945). Polymorphism. Biol Rev.

[5] Clarke B (1960). Divergent effects of natural selection on two closely-related polymorphic snails. Heredity.

[6] Hof AE, Campagne P, Rigden DJ, Yung CJ, Lingley J, Quail MA, Hall N, Darby AC, Saccheri IJ (2016). The industrial melanism mutation in british peppered moths is a transposable element. Nature.

[7] Eakley AL, Houde AE (2004). Possible role of female discrimination against ‘redundant’ males in the evolution of colour pattern polymorphism in guppies. Proc R Soc B.

[8] Davis Rabosky AR, Cox CL, Rabosky DL, Title PO, Holmes IA, Feldman A, McGuire JA. Coral snakes predict the evolution of mimicry across new world snakes. Nat Commun. 2016;7:1–9. 10.1038/ncomms11484 PMC4858746 27146100

[9] Mallet J, Joron M (1999). Evolution of diversity in warning color and mimicry: polymorphisms, shifting balance, and speciation. Ann Rev Ecol Syst..

[10] Nadeau N, Pardo-Diaz C, Whibley A, Supple MA, Saenko SV, Wallbank RWR, Wu GC, Maroja L, Ferguson L, Hanly JJ, Hines H, Salazar C, Merrill RM, Dowling AJ, ffrench-Constant RH, Llaurens V, Joron M, WO MM, Jiggins CD (2016). The gene cortex controls mimicry and crypsis in butterflies and moths. Nature.

[11] Clarke C, Sheppard P (1960). The evolution of mimicry in the butterfly *Papilio dardanus*. Heredity.

[12] Endler JA (1993). The color of light in forests and its implications. Ecol Monogr.

[13] Cronin TW, Johnsen S, Marshall NJ, Warrant EJ. Visual ecology. 1st ed. Princeton: Princeton University Press; 2014.

[14] White TE, Zeil J, Kemp DJ (2015). Signal design and courtship presentation coincide for highly biased delivery of an iridescent butterfly mating signal. Evolution.

[15] Sicsu P, Manica LT, Maia R, Macedo RH (2013). Here Comes the sun: multimodal displays are associated with sunlight incidence. Behav Ecol Sociobiol.

[16] Barry KL, White TE, Rathnayake DN, Fabricant SA, Herberstein ME (2015). Sexual signals for the colour-blind: cryptic female mantids signal quality through brightness. Fun Ecol.

[17] Uy JAC, Endler JA (2004). Modification of the visual background increases the conspicuousness of golden-collared manakin displays. Behav Ecol.

[18] McKaye KR, Louda SM, Stauffer JR Jr. Bower size and male reproductive success in a cichlid fish lek. Am Nat. 1990:597–613.

[19] Lythgoe JN. Ecology of vision. Clarendon Press; Oxford University Press; 1979.

[20] Endler JA (1992). Signals, signal conditions, and the direction of evolution. Am Nat.

[21] Fuller RC (2002). Lighting environment predicts the relative abundance of male colour morphs in bluefin killifish (*Lucania goodei*) populations. Proc R Soc B.

[22] Gomez D, Théry M (2004). Influence of ambient light on the evolution of colour signals: comparative analysis of a neotropical rainforest bird community. Ecol Lett.

[23] Stuart-Fox D, Moussalli A, Whiting MJ (2007). Natural selection on social signals: signal efficacy and the evolution of chameleon display coloration. Am Nat.

[24] Chunco AJ, McKinnon JS, Servedio MR (2007). Microhabitat variation and sexual selection can maintain male color polymorphisms. Evolution.

[25] Rojas B, Rautiala P, Mappes J (2014). Differential detectability of polymorphic warning signals under varying light environments. Behav Proc.

[26] White TE, Kemp DJ (2015). Technicolour deceit: a sensory basis for the study of colour-based lures. Anim Behav.

[27] Osorio D, Vorobyev M (2005). Photoreceptor spectral sensitivities in terrestrial animals: adaptations for luminance and colour vision. Proc R Soc B.

[28] Hart NS (2001). The visual ecology of avian photoreceptors. Prog Ret Eye Res.

[29] de Ibarra NH, Vorobyev M, Menzel R. Mechanisms, functions and ecology of colour vision in the honeybee. J Comp Phys A. 2014:1–23. 10.1007/s00359-014-0915-1 PMC4035557 24828676

[30] Menzel R, Backhaus W. Color vision honey bees. In: Stavenga DG, Hardie RC, editors. Phenomena and physiological mechanisms: Facets of vision, Springer; 1989. p. 281–97.

[31] de Ibarra NH, Giurfa M, Vorobyev M (2001). Detection of coloured patterns by honeybees through chromatic and achromatic cues. J Comp Phys A.

[32] Osorio D, Miklósi A, Gonda Z (1999). Visual ecology and perception of coloration patterns by domestic chicks. Evol Ecol.

[33] Giurfa M, Vorobyev M, Brandt R, Posner B, Menzel R (1997). Discrimination of coloured stimuli by honeybees: alternative use of achromatic and chromatic signals. J Comp Phys A.

[34] Jones C, Osorio D (2004). Discrimination of oriented visual textures by poultry chicks. Vis Res.

[35] White TE, Kemp DJ (2016). Colour polymorphic lures target different visual channels in prey. Evolution.

[36] White TE. Jewelled spiders manipulate colour lure geometry to deceive prey. Biol Lett. 2017;20170027. 10.1098/rsbl.2017.0027 PMC5377040 28356411

[37] Kemp DJ, Holmes C, Congdon BC, Edwards W. Color polymorphism in spiny spiders (*Gasteracantha fornicata*): testing the adaptive significance of a geographically clinal lure. Ethology. 2013:1126–37.

[38] Lunau K, Maier EJ (1995). Innate colour preferences of flower visitors. J Comp Phys A.

[39] Burg J, Axtell R (1984). Monitoring house fly, *Musca domestica* (diptera: Muscidae), populations in caged-layer poultry houses using a baited jug-trap. Enviro Ento..

[40] Riedl H, Hislop R (1985). Visual attraction of the walnut husk fly (diptera: Tephritidae) to color rectangles and spheres. Enviro Ento.

[41] Campbell DR, Bischoff M, Lord JM, Robertson AW (2010). Flower color influences insect visitation in alpine new zealand. Ecology.

[42] Lunau K. Visual ecology of flies with particular reference to colour vision and colour preferences. J Comp Phys A. 2014:1–16. 10.1007/s00359-014-0895-1 24664124

[43] Giurfa M, Vorobyev M, Kevan P, Menzel R (1996). Detection of coloured stimuli by honeybees: minimum visual angles and receptor specific contrasts. J Comp Phys A..

[44] Vorobyev M, Osorio D (1998). Receptor noise as a determinant of colour thresholds. Proc R Soc B.

[45] Dyer AG, Chittka L (2004). Fine colour discrimination requires differential conditioning in bumblebees. Naturvis.

[46] Dyer AG, Chittka L (2004). Bumblebees (*Bombus terrestris*) sacrifice foraging speed to solve difficult colour discrimination tasks. J Comp Phys A..

[47] Maia R, Eliason CM, Bitton PP, Doucet SM, Shawkey MD. Pavo: an r package for the analysis, visualization and organization of spectral data. Methods Ecol Evol. 2013:906–13.

[48] Core Team R (2014). R: a language and environment for statistical computing.

[49] Vowles D (1966). The receptive fields of cells in the retina of the housefly (*Musca domestica*). Proc R Soc B.

[50] Stavenga DG (2003). Angular and spectral sensitivity of fly photoreceptors. i. Integrated facet lens and rhabdomere optics. J Comp Phys A.

[51] Gonzalez-Bellido PT, Wardill TJ, Juusola M (2011). Compound eyes and retinal information processing in miniature dipteran species match their specific ecological demands. PNAS.

[52] Maimon G, Straw AD, Dickinson MH (2008). A simple vision-based algorithm for decision making in flying *Drosophila*. Curr Biol.

[53] Sokal RR (1995). Biometry: the principles and practice of statistics in biological research.

[54] Endler JA. Natural selection in the wild: Princeton University Press; 1986.

[55] Hedrick PW, Ginevan ME, Ewing EP. Genetic polymorphism in heterogeneous environments. Ann Rev Ecol Syst. 1976:1–32.

[56] Gray SM, Dill LM, Tantu FY, Loew ER, Herder F, McKinnon JS (2008). Environment-contingent sexual selection in a colour polymorphic fish. Proc R Soc B.

[57] Tso IM, Lin CW, Yang EC (2004). Colourful orb-weaving spiders, *Nephila pilipes*, through a bee’s eyes. J Exp Biol.

[58] Heiling AM, Chittka L, Cheng K, Herberstein ME (2005). Colouration in crab spiders: substrate choice and prey attraction. J Exp Biol.

[59] Llandres AL, Gawryszewski FM, Heiling AM, Herberstein ME (2011). The effect of colour variation in predators on the behaviour of pollinators: Australian crab spiders and native bees. Ecol Ento.

[60] Chiao CC, Wu WY, Chen SH, Yang EC (2009). Visualization of the spatial and spectral signals of orb-weaving spiders, *Nephila pilipes*, through the eyes of a honeybee. J Exp Biol.

[61] Chuang CY, Yang EC, Tso IM (2008). Deceptive color signaling in the night: a nocturnal predator attracts prey with visual lures. Behav Ecol.

[62] Troje N (1993). Spectral categories in the learning behaviour of blowflies. Zeits Natur.

[63] Fukushi T (1994). Colour perception of single and mixed monochromatic lights in the blowfly *Lucilia cuprina*. J Comp Phys A.

[64] Menne D, Spatz HC (1977). Colour vision in *Drosophila melanogaster*. J Comp Phys A..

[65] Salomon CH, Spatz HC (1983). Colour vision in *Drosophila melanogaster*: wavelength discrimination. J Comp Phys A.

[66] Endler JA, Gaburro J, Kelley LA. Visual effects in great bowerbird sexual displays and their implications for signal design. Proc R Soc B. 2014;20140235. 10.1098/rspb.2014.0235 PMC3996615 24695430

[67] Kelber A (2005). Alternative use of chromatic and achromatic cues in a hawkmoth. Proc R Soc B.

[68] Schaefer HM, Levey DJ, Schaefer V, Avery ML (2006). The role of chromatic and achromatic signals for fruit detection by birds. Behav Ecol.

[69] White TE, Dalrymple RL, Herberstein ME, Kemp DJ. The perceptual similarity of flower colours and prey lures. Evol Ecol. 2017;1:1–20.

