# Supplementary material for: Colour polymorphic lures exploit innate preferences for spectral versus luminance cues in dipteran prey
Source: BMC Evol Biol. 2017 Aug 14;17:191. doi: 10.1186/s12862-017-1043-7 (PMC5557064; doi:10.1186/s12862-017-1043-7)
Supplement: Additional file 1: — Additional experiment: details the methods of the additional experiment. Figure S1 depicts the Y maze apperatus for choice assays. Figure S2 shows the reflectance spectra of stimuli used in the additional choice experiment. Figure S3 presents the results of the supplementary experiment. Table S1 denotes the approximate chromatic (unitless) and achromatic (Michelson) target/background contrast of model ‘colourful and ‘luminant’ stimuli from Additional experiment S1, as modelled according to the visual systems of D.melanogaster and M. domestica. (PDF 329 kb) [file 12862_2017_1043_MOESM1_ESM.pdf]

Electronic supplementary material:

Colour polymorphic lures exploit innate preferences for spectral versus luminance cues in dipteran prey.

Supplementary experiment: innate preferences for colour versus luminance cues

We conducted a supplementary experiment to test the assumption that *D. hydei* and *M. domestica* vary their preference for colour and luminance cures as a function of the apparent stimulus size, using an assay similar to those detailed for our main experiments (see main text). We simultaneously presented colour-naive flies (n = 80 of both species per condition) with two target/background combinations: one that generated strong chromatic contrast, but relatively little luminance contrast, and one that generated strong luminance contrast but relatively little chromatic contrast (Fig. S2; Table S1). The targets were circular cardboard sheets (Elle card) of 200 mm diameter, which were fixed at the centre of 250 x 300 mm backgrounds. We repeated the assay twice, varying the apparent size of models so that they subtended visual angles of either 10° or 50°. If flies alternate their reliance on colour and luminance cues for target identification, we predicted that they should prefer the 'chromatic' stimulus when stimuli were presented at the relatively large visual angle of 50°, and the 'achromatic' stimulus when presented at the relatively small visual angle of $10^{\,o}$.

Binomial tests for equal-choice frequency support this prediction, and show that both *M. domestica* (*N* = 52/80, *P* = 0.01) and *D. hydei* (*N* = 62/80, *P* = < 0.01) preferred the chromatic stimulus when the targets subtended a visual angle of 50° (Fig. S2). Conversely, the innate preferences of both *M. domestica* (*N* = 57/80, *P* = < 0.01) and *D. hydei* (*N* = 51/80, *P* = 0.01) shifted to the achromatic stimulus when presented at 10°. This is only a tentative result of course, and truly detailing the dynamic use of colour and luminance channels in flies would require extensive testing with trained animals and tightly controlled stimuli. It is, nonetheless, consistent with the notion that the innate use of colour and luminance cues in these flies shifts as a function of apparent stimulus size; a seemingly general feature of many diurnally-adapted visual systems (see main text for further discussion).

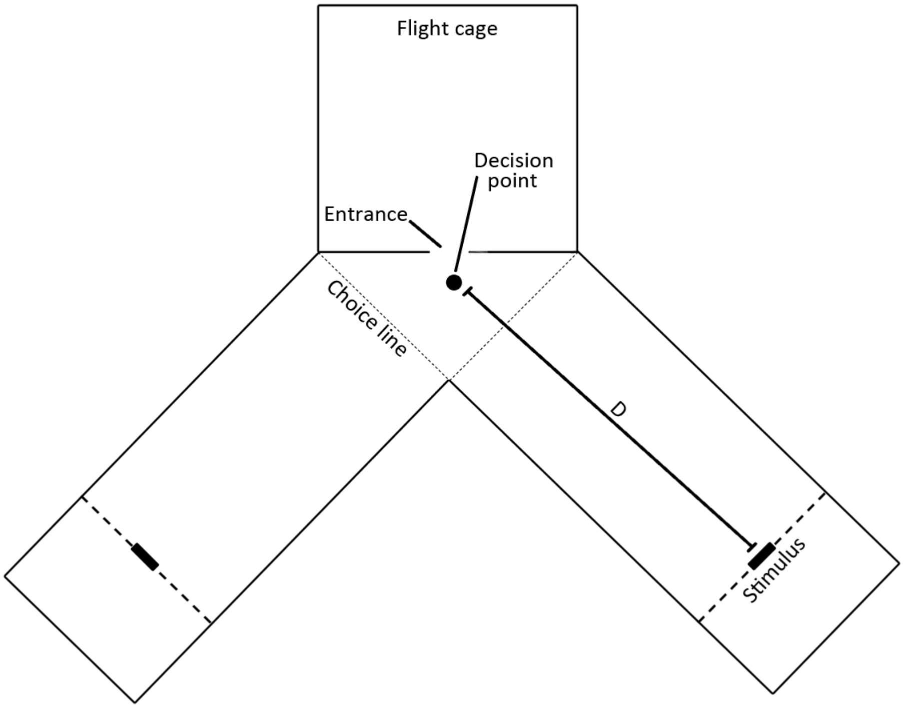

Figure S1: The Y maze apperatus for choice assays. Individual, colour-naive flies entered via the flight cage and simultaneously viewed both maze arms. A decision was counted when a fly crossed the choice line (dashed). We varied the distance between the stimuli and the maze's 'choice point' (D) to control the visual angle subtended by the models (calculated using the left-right axis of model spiders) in nine increments; 1°, 6°, 11°, 16°, 21°, 26°, 31°, 36°, and 41°.

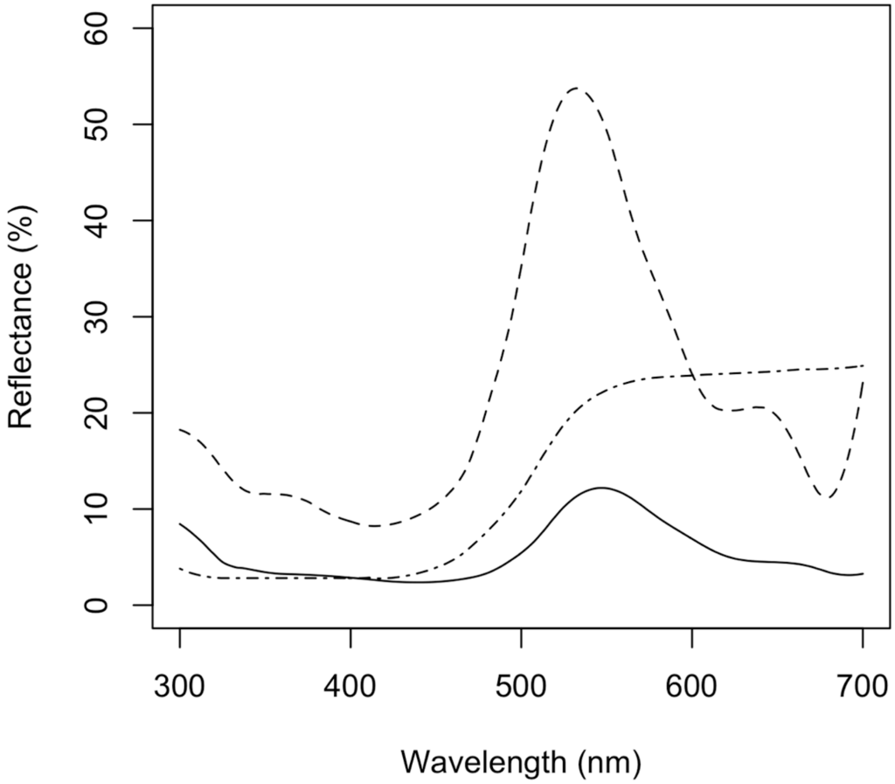

Figure S2: Reflectance spectra of stimuli used in the supplementary choice experiment. For the 'chromatic' stimulus, a yellow (dot-dash line) target was presented against a dark green background (solid line), while the 'achromatic' stimulus comprised a light green (dashed line) target against the same dark green background.

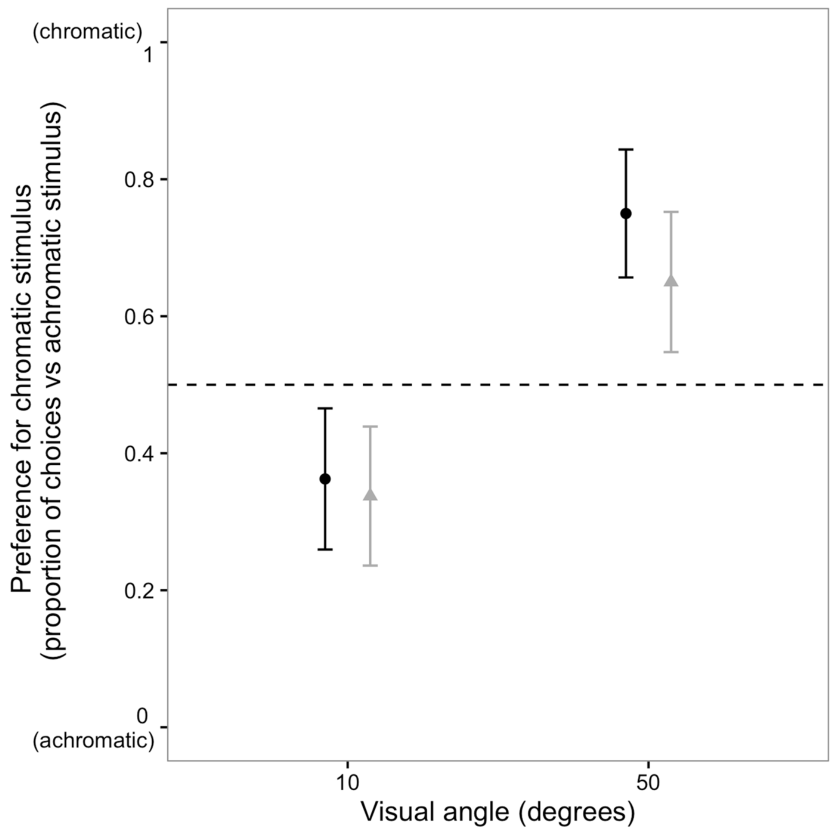

Figure S3: The proportion of innate choices by *D. hydei* (dark bars) and *M. domestica* (light bars) for stimuli that generate strong chromatic versus achromatic contrast, as a function of the apparent model size (visual angle subtended by stimuli at the choice point).

Table S1: The broadly estimated chromatic (unitless) and achromatic (Michelson) target/background contrast of model 'colourful and 'luminant' stimuli from supplementary experiment S1, as modelled according to the visual systems of *D. melanogaster* and *M. domestica* (supplementary methods, below).

		**Stimulus**	
**viewer**	**vis. contrast**	**colourful**	**luminant**
D. melanogaster	chromatic	0.39	0.10
	achromatic	0.08	0.61
M. domestica	chromatic	0.41	0.12
	achromatic	0.10	0.62

Supplementary methods: Estimating colour and luminance contrast to dipteran viewers

We used a model of fly vision to broadly estimate the visual effects induced by our experimental manipulations. Given the uncertainty surrounding models of colour and luminance sensation in Diptera, these models were not strongly relied upon for the design of our experimental stimuli (which were instead informed by an extensive natural spectral dataset; White & Kemp 2016). We used the visual phenotypes of *Drosophila melanogaster* and *Musca domestica* when estimating the magnitude of chromatic and achromatic model/background contrasts (Salcedo et al. 1999). For chromatic contrasts, we first calculated receptor quantum catches as:

$$Q_i = \int_{300}^{700} R(\lambda)I(\lambda)S_i(\lambda)d\lambda$$

where *R* is the spectral reflectance of the spider model or background, *I* is the illuminating spectrum, and S_i is the spectral sensitivity of photoreceptor *i*. We then normalized the quantum catch data to sum to one, and calculated the difference in relative stimulation between the 'pale' and 'yellow' photoreceptor pairs (Troje 1993).

$$x = Q_{R7p} - Q_{R8p}$$

$$y = Q_{R7y} - Q_{R8y}$$

Chromatic contrast was then estimated as the Euclidean distance (unitless) between points (Shrestha et al. 2016):

$$\sqrt{(x_1 - x_2)^2 + (y_1 - y_2)^2}$$

Luminance (Michelson) contrast was calculated as:

$$\frac{Q_s - Q_b}{Q_s + Q_b}$$

where Q_s and Q_b refer to the quantum catches (equation 1) of each species' R1-6 photoreceptor when viewing the spiders and backgrounds, respectively (Salcedo et al. 1999; Fleishman et al. 2001).
